# LncRNA GAS5 participates in childhood pneumonia by inhibiting cell apoptosis and promoting SHIP-1 expression via downregulating miR-155

**DOI:** 10.1186/s12890-021-01724-y

**Published:** 2021-11-11

**Authors:** Xiaoping Wang, Ping Guo, Jiahui Tian, Jie Li, Na Yan, Xin Zhao, Yue Ma

**Affiliations:** Department of Respiratory and Critical Medicine, Clinical Medical College of Hulunbeier, Inner Mongolia University for Nationalities, Hulunbuir People’s Hospital, Hulunbuir City, 021008 Inner Mongolia People’s Republic of China

**Keywords:** GAS5, Childhood pneumonia, miR-155, Apoptosis, SHIP-1, Methylation

## Abstract

**Background:**

LncRNA GAS5 and miR-155 are reported to play opposite roles in lung inflammatory responses. Lung inflammation participates in childhood pneumonia, indicating the involvement of GAS5 and miR-155 in pneumonia. The study aimed to analyze the potential interaction between GAS5 and miR-155 in childhood pneumonia.

**Methods:**

GAS5 and miR-155 levels in plasma samples from pneumonia patients and controls were detected using RT-qPCR. The role of GAS5 in miR-155 RNA gene methylation in human bronchial epithelial cells (HBEpCs) was analyzed by methylation analysis. Flow cytometry and RT-qPCR were applied to analyze cell apoptosis and SHIP-1 expression, respectively.

**Results:**

GAS5 was downregulated in pneumonia, and miR-155 was upregulated in pneumonia. GAS5 and miR-155 were inversely correlated. GAS5 overexpression decreased miR-155 expression in HBEpCs, while miR-155 overexpression showed no significant effects on GAS5 expression. In addition, GAS5 suppressed LPS-induced HBEpC apoptosis, promoted SHIP-1 expression, and reduced the enhancing effect of miR-155 on cell apoptosis and SHIP-1 expression.

**Conclusions:**

GAS5 may participate in childhood pneumonia by inhibiting cell apoptosis and promoting SHIP-1 expression via downregulating miR-155.

**Supplementary Information:**

The online version contains supplementary material available at 10.1186/s12890-021-01724-y.

## Introduction

Pneumonia is the infection of one or both lungs caused by viruses, bacteria, and fungi [1, 2] and is very common in children [3]. It affects about 0.28 episodes per child-year or annually affects more than 150 million children. Among them, 11–20 million (7–13%) are severe cases requiring hospitalization [3]. Unfortunately, about 5–10% of hospitalized cases will die of pneumonia within 30 days after admission [4, 5]. Pneumonia is usually treated with macrolide antibiotics, fluoroquinolones, and tetracyclines [6, 7] with satisfactory outcomes in general. However, sides effects are not avoidable, leading to poor prognosis [6, 7].

Besides infections, previous studies have shown the participation of molecular players in pneumonia [8, 9]. Functional analysis of these factors may improve the treatment of pneumonia [8, 9]. NcRNAs do not directly encode proteins, but they regulate the expression of non-coding RNA genes and coding genes. Thus, targeting the expression of ncRNAs may assist the recovery of certain diseases [10, 11]. However, the functions of most ncRNAs in human diseases such as pneumonia remain unclear. Previous studies have shown that lncRNA GAS5 and miR-155 play opposite roles in responses to lung inflammation [12, 13], a key player in pneumonia [14], suggesting the involvement of GAS5 and miR-155 in pneumonia. Thus, we analyzed the potential interaction between GAS5 and miR-155 in pneumonia.

Src homology 2-containing inositol phosphatase-1 (SHIP-1) is a target of miR-155, a pro-inflammatory factor (PMID: 31907997) [1]. SHIP-1 gene deletion in mice leads to an immunological phenotype with overproduction of pro-inflammatory cytokines, activation of myeloid cells, and severe inflammation in the lung [15, 16]. Therefore, we also explored the interactions of SHIP-1 with GAS5 and miR-155 in pneumonia.

## Materials and methods

### Research subjects

The study was approved by the Ethics Committee of our hospital and included both childhood pneumonia (CP) patients (n = 62, 37 males and 25 females, 1–3 years, 1.7 ± 0.5 years) and healthy controls (n = 62, 37 males and 25 females, 1–3 years, 1.7 ± 1.4 years) who were enrolled at Inner Mongolia University for Nationalities, Hulunbuir People's Hospital between March 2018 and March 2020. The pneumonia was caused by either viral (n = 34) or bacterial (n = 28) infections. CP was defined according to the World Health Organization’s guidelines for acute respiratory infection [2, 17]. All CP patients met the following criteria: (1) symptoms of cough, fever, abnormal breathing, and fixed medium and small alveolar sounds in the lung; (2) increased neutrophils (> 8 × 10^9^/L) and PLTs (> 300 × 10^9^/L); and (3) patchy shadows observed by Chest X-ray. Patients with chronic renal or hepatic diseases, hematological diseases, inflammatory bowel diseases, chronic obstructive pulmonary diseases, asthma, SARS-CoV2, and congenital diseases and patients who had received anti-inflammatory treatment were excluded from this study. All the 62 healthy controls received a systemic-level physiological examination at our hospital during the same time period. Table 1 shows the clinical data of patients and controls. Informed consent was obtained from all individual participants or their guardians. Procedures operated in this research were completed in keeping with the standards set out in the Announcement of Helsinki and Laboratory Guidelines of Research in China.

**Table 1:** Clinical data of patients and controls

	Control (n = 62)	Pneumonia (n = 62)
Years	1.7 ± 1.4	1.7 ± 0.5
RBC (× 10^12^/L)	4.99 ± 0.43	3.43 ± 0.67
WBC (× 10^9^/L)	6.92 ± 1.72	11.23 ± 2.78
PLT (× 10^9^/L)	267.81 ± 70.12	203.43 ± 61.98
HB (g/L)	134.62 ± 18.59	109.52 ± 20.53
HCT	0.43 ± 0.043	0.38 ± 0.033
DD (mg/L)	3.11 ± 0.87	5.98 ± 1.01
PaO_2_ (mmHg)	86.37 ± 14.12	57.45 ± 18.23
PaCO_2_ (mmHg)	40.39 ± 9.77	34.89 ± 7.48

Red blood cell (RBC), white blood cell (WBC), platelet count (PLT), Hemoglobin (HB), hematocrit (HCT), D-dimer (DD), Partial arterial oxygen pressure (PaO2), partial pressure of carbon dioxide in artery (PaCO2)

### Preparation of plasma samples

Fasting venous blood samples (5 ml) were collected from patients the next morning after hospitalization and from healthy controls in EDTA tubes and centrifuged at 1200*g* for 10 min at room temperature to separate the plasma. RNAs were extracted within 6 h after plasma preparations (Table 2).

**Table 2:** Correlation of GAS5 and miR-155 expression with clinicopathologic parameters in 62 Pneumonia patients

	Pneumonia (n = 62)	GAS5 expression level		*P* value	miR-155 expression level		*P* value
		Low	High		Low	High	
Years							
≤ 2	30	14	16	*P* > 0.05	18	12	*P* > 0.05
> 2	32	17	15	*P* > 0.05	16	16	*P* > 0.05
Gender							
Male	37	16	21	*P* > 0.05	19	18	*P* > 0.05
Female	25	13	12	*P* > 0.05	10	15	*P* > 0.05
Infection							
Viral	34	16	18	*P* > 0.05	17	17	*P* > 0.05
Bacterial	28	15	13	*P* > 0.05	12	16	*P* > 0.05
PSI							
I–III	16	4	12	*P* < 0.05**	11	5	*P* < 0.05**
IV	28	18	10	*P* < 0.01**	8	20	*P* < 0.01**
V	18	16	2	*P* < 0.01**	1	17	*P* < 0.01**

Pneumonia severity index (PSI), *P < 0.05, **P < 0.01

### Bronchoalveolar lavage (BAL)

Alveolar lavage was performed with sterile normal saline (0.3–0.5 mL/kg) following the guidelines described previously [18] to collect bronchoalveolar lavage fluid (BALF) from patients and healthy controls. Within 1 h after BALF collection, supernatants were separated, aliquoted, and stored at − 20 °C.

### Human bronchial epithelial cells (HBEpC)

HBEpCs were cultured in Bronchial Epithelial Cell Medium (ScienCell) or RPMI-1640 medium supplemented with 10% fetal bovine serum (FBS) at 37 °C in an incubator with 5% CO_2_. Cells at passages 4–6 were used for subsequent experiments. For LPS treatment, cells were cultured in media containing 0, 5, and 10 ng/ml LPS for 48 h. For 5-azacytidine (5-azaC, Sigma-Aldrich) treatment, HBEpCs were incubated in media containing 1, 10, or 100 nM 5-azaC for 24 h.

### Vectors, miRNAs, and transfections

To overexpress GAS5, the full length of GAS5 was synthesized by GenePharma (Shanghai, China) and cloned into pcDNA3.1 vector (GenePharma, Shanghai, China) to generate GAS5 overexpressing vector named GAS5. MiR-155 mimic and negative control (NC) miRNA were purchased from Sangon (Shanghai, China). Expression vector (1 μg) or miRNA (50 nM) was transfected into HBEpCs using Lipofectamine 2000 (Invitrogen). Empty vector or NC miRNA transfected cells were served as NC groups. Untransfected cells were used as control (C) cells. At 48 h post-transfection, cells were subjected to subsequent experiments.

### RNA samples

Total RNAs were extracted from plasma samples and HBEpCs using Ribozol (Invitrogen) and treated with DNase I (Sangon) for 2 h at 37 °C to remove DNA. RNA integrity was checked on 5% urine-PAGE gels.

### Real-time quantitative PCR (RT-qPCR)

cDNA samples were synthesized using QuantiTect Reverse Transcription Kits (QIAGEN). After that, qPCRs were performed with cDNA samples as templates using SYBR Green Master Mix (Bio-Rad) to measure the levels of GAS5 and SHIP-1 with GAPDH as the internal control. To determine the mature miR-155 level, poly (A) addition, reverse transcriptions, and qPCRs were sequentially performed using GeneCopoeia All-in-One™ miRNA qRT-PCR Reagent Kit. The method of 2^−ΔΔCt^ was used for Ct value normalizations. The primer sequences were GAS5 forward 5′-GCTTACTGCTTGAAAGGGTCT-3′ and reverse 5′-CACTGGGAGGCTGAGGAT-3′, miR-155 forward 5′-GGGGGTAATGCTAATCGTGAT-3′ and reverse 5′-GTGC GTGTCGTGGAGTCG-3′; U6 forward 5′-CTCGCTTCGGCAGCACA-3′ and reverse 5′-AACGCTTCACGAATTTGCGT-3′; SHIP-1 forward 5′-CAGGGATGAAGTA CAACTTGCC-3′ and reverse 5′-TCTCCTTCCTGACTCTTGACA-3′; GAPDH forward 5′-CGGACCAATACGACCAAATCCG-3′ and reverse 5′-AGCCACATCGC TCAGACACC-3′.

### Methylation-specific polymerase chain reaction (MSP)

Genomic DNAs were extracted using DNeasy Tissue Kits (Qiagen) following manufacturer’s instructions. EZ DNA Methylation Lighting Kit was used to convert DNA. Routine PCR and MSP were performed, and PCR products were separated using 2% agarose gel electrophoresis. Ultraviolet irradiation was used to visualize the bands. The primer sequences were methylation forward 5′-TAGTCGATTGAAAGTTCGG GC-3′ and methylation reverse 5′-CCTTTCTCGTAAATCATTAC-3′, and unmethylation forward 5′-TTTTAGTTGATTGAAAGTTTGGGT-3′ and unmethylation reverse 5′-TTTCCCTTTCTCATAAATCATTACA-3′.

### Apoptosis assay

Cells were cultured in media containing 10 ng/ml LPS for 48 h. After that, cells were fixed using 70% ethanol and stained with PI and Annexin-V FITC. FITC-IgG1 was used as an isotype control. At last, apoptotic cells were analyzed by FACS Calibur instrument.

### Statistical analysis

Three independent replicates were included in each experiment. All data were expressed as mean ± SD values. Unpaired t test was used to compare patient and control groups. Multiple cell transfection groups were compared by ANOVA Tukey’s test. P < 0.05 was statistically significant.

## Results

### GAS5 and miR-155 expression was altered in pneumonia

**Fig. 1:**
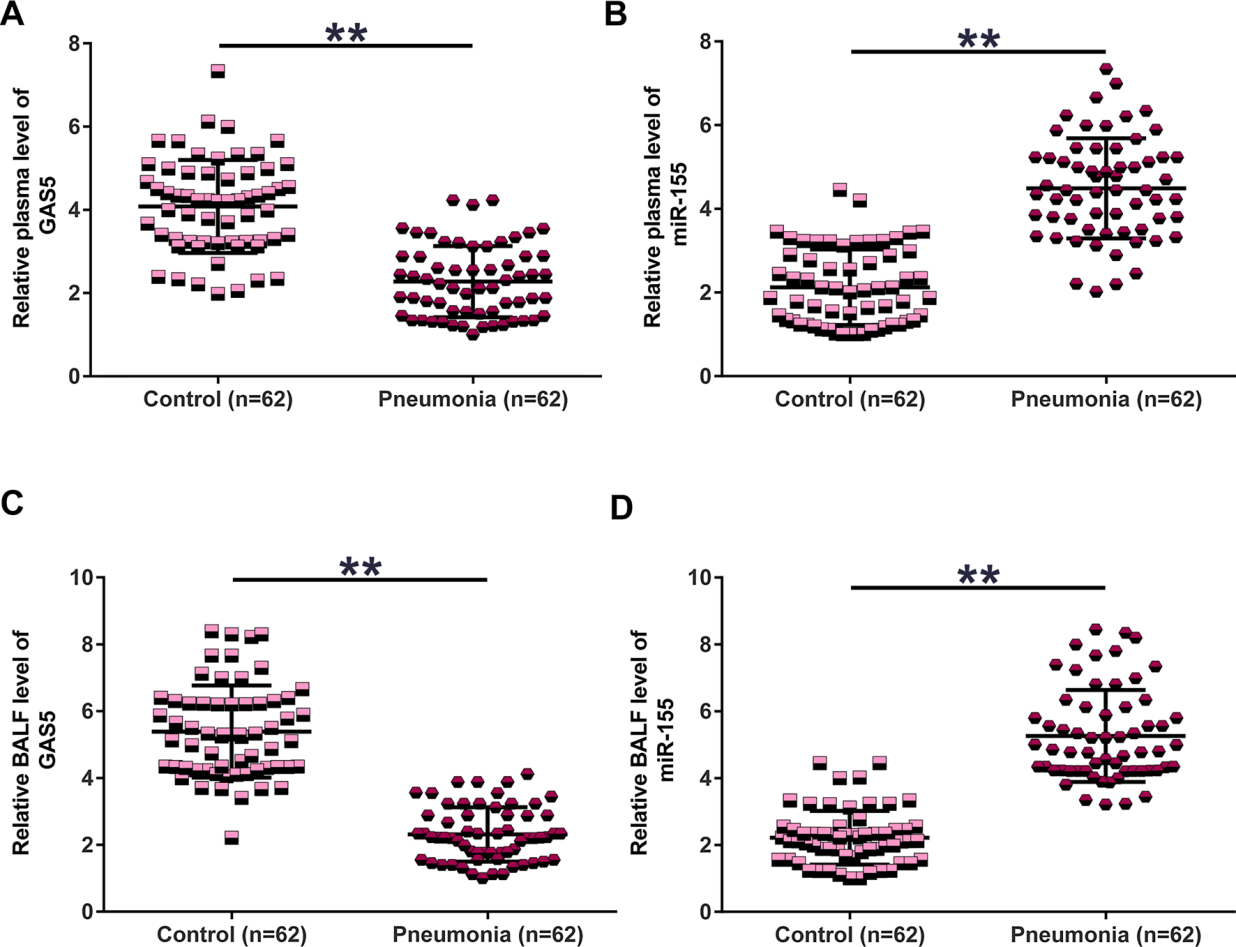
GAS5 and miR-155 expression was altered in pneumonia. GAS5 (**a**) and miR-155 (**b**) expression levels in plasma samples from both pneumonia patients (n = 62) and healthy controls (n = 62) were determined by RT-qPCR. GAS5 (**c**) and miR-155 (**d**) expression levels in BALF samples from both pneumonia patients (n = 62) and healthy controls (n = 62) were determined by RT-qPCR. Unpaired t test was used to compare patient and control groups. **P < 0.01

GAS5 and miR-155 expression levels in plasma samples from both pneumonia (n = 62) and healthy controls (n = 62) were analyzed. RT-qPCR experiments illustrated that plasma GAS5 level was downregulated in pneumonia patients (Fig. 1a, *P* < 0.01). In contrast, plasma miR-155 level was significantly higher in the pneumonia group than in the control group (Fig. 1b, *P* < 0.01). In addition, GAS5 and miR-155 mRNA levels in BALF were analyzed in both pneumonia (n = 62) and healthy controls (n = 62) using RT-qPCR. Similarly, GAS5 expression was downregulated (Fig. 1c, *P* < 0.01), and miR-155 expression was upregulated (Fig. 1d *P* < 0.01) (Fig. 1a, *P* < 0.01) in BALF of pneumonia group than in the control group.

### GAS5 and miR-155 were closely correlated with each other

**Fig. 2:**
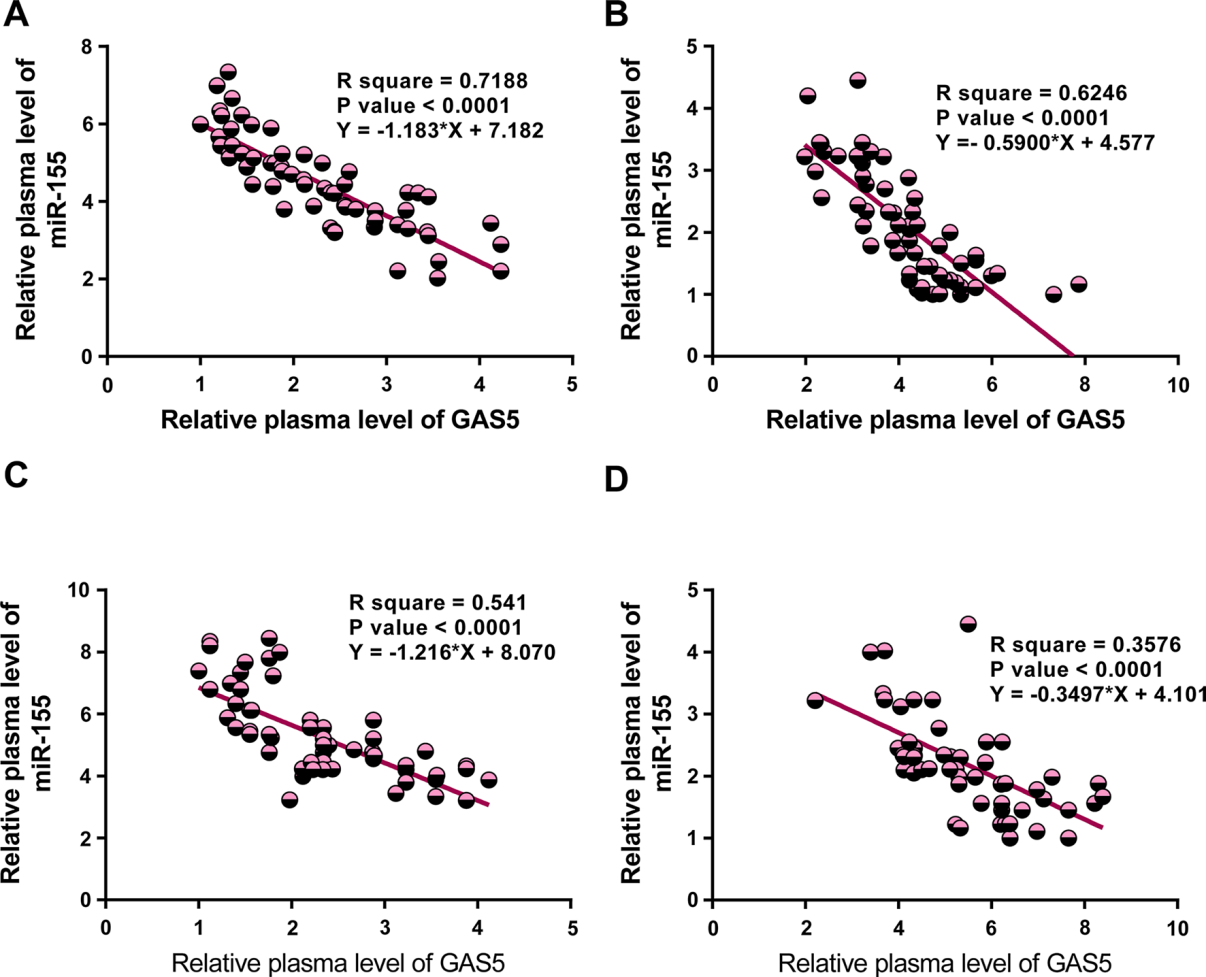
Correlation analysis between GAS5 and miR-155. Correlations between plasma GAS5 and miR-155 levels across both pneumonia samples (**a**) and control samples (**b**) and between BALF GAS5 and miR-155 levels across both pneumonia samples (**c**) and control samples (**d**) were analyzed by linear regression

Correlations between GAS5 and miR-155 levels in plasma and BALF across both pneumonia samples and control samples were analyzed by linear regression. As shown in Fig. 2, GAS5 and miR-155 were inversely correlated across both pneumonia samples and control samples.

### GAS5 overexpression decreased miR-155 expression in HBEpCs via methylation

**Fig. 3:**
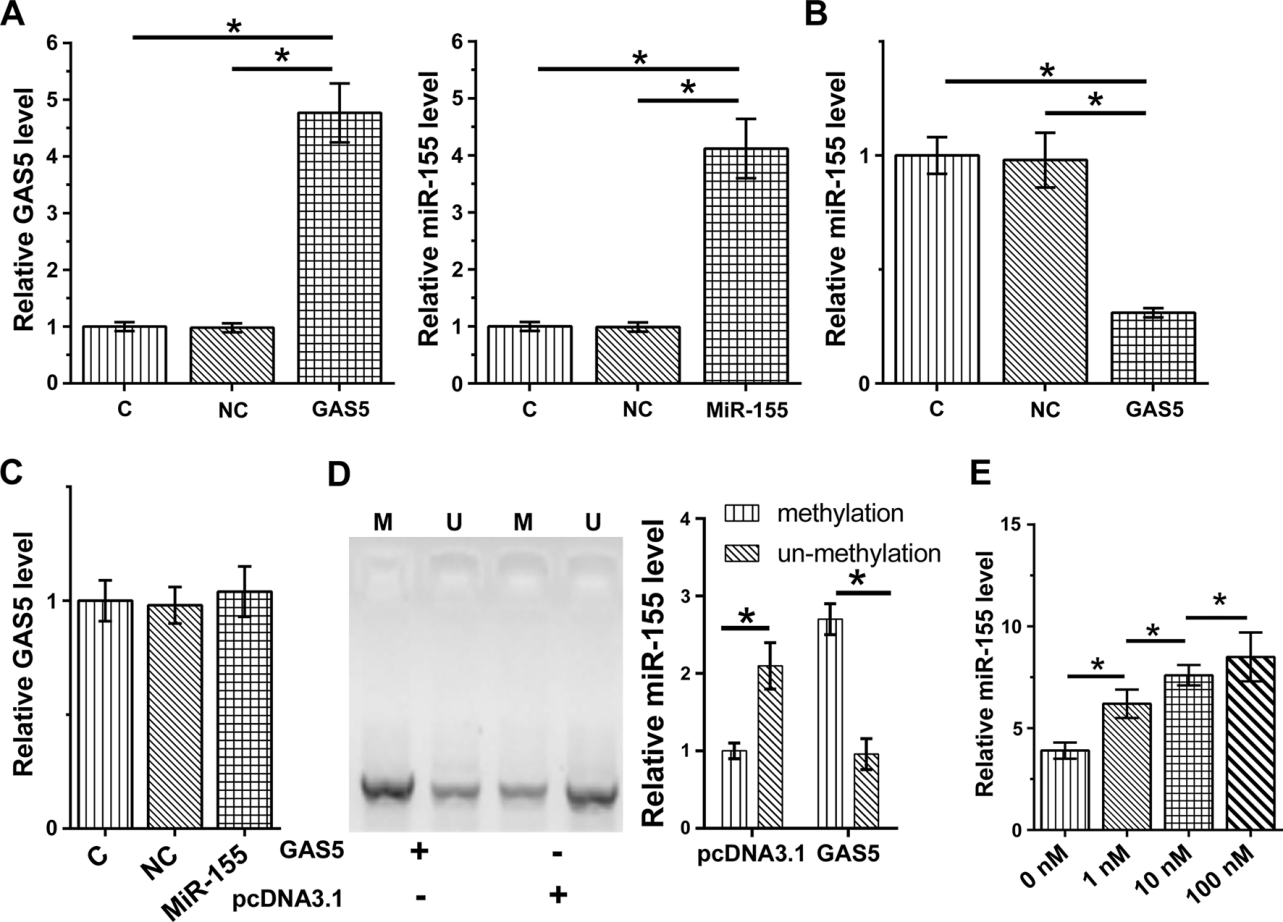
The interaction between GAS5 and miR-155 in HBEpCs. GAS5 and miR-155 were overexpressed in HBEpCs through overexpression (**a**). The role of GAS5 in regulating miR-155 expression (**b**) and the role of miR-155 in regulating GAS5 expression (**c**) was studied by RT-qPCR. MSP was performed to analyze the effects of GAS5 overexpression on miR-155 methylation. **d** HBEpCs were incubated in media containing 1, 10, or 100 nM 5-azaC for 24 h, and miR-155 expression was analyzed by RT-qPCR. Data were derived from three independent experiments, expressed as mean ± SD, and compared by ANOVA Tukey’s test. * P < 0.05

HBEpCs were overexpressed with GAS5 and miR-155 to analyze the crosstalk between them (Fig. 3a, *P* < 0.05). Interestingly, GAS5 overexpression decreased miR-155 expression (Fig. 3b, *P* < 0.05), while miR-155 overexpression showed no significant effects on GAS5 expression (Fig. 3c). MSP experiments illustrated that miR-155 RNA gene methylation was increased in cells transfected with GAS5 expression vector (Fig. 3d). Moreover, HBEpCs were treated for 24 h with 1, 10, or 100 nM 5-azaC, a methylation inhibitor. These treatments significantly upregulated miR-155 expression level in a dose-dependent manner compared to the control group (Fig. 3e) (*P* < 0.05), indicating that GAS5 might downregulate miR-155 by increasing its methylation.

### GAS5 suppressed HBEpC apoptosis through miR-155 and increased the expression of SHIP-1, a target of miR-503

**Fig. 4:**
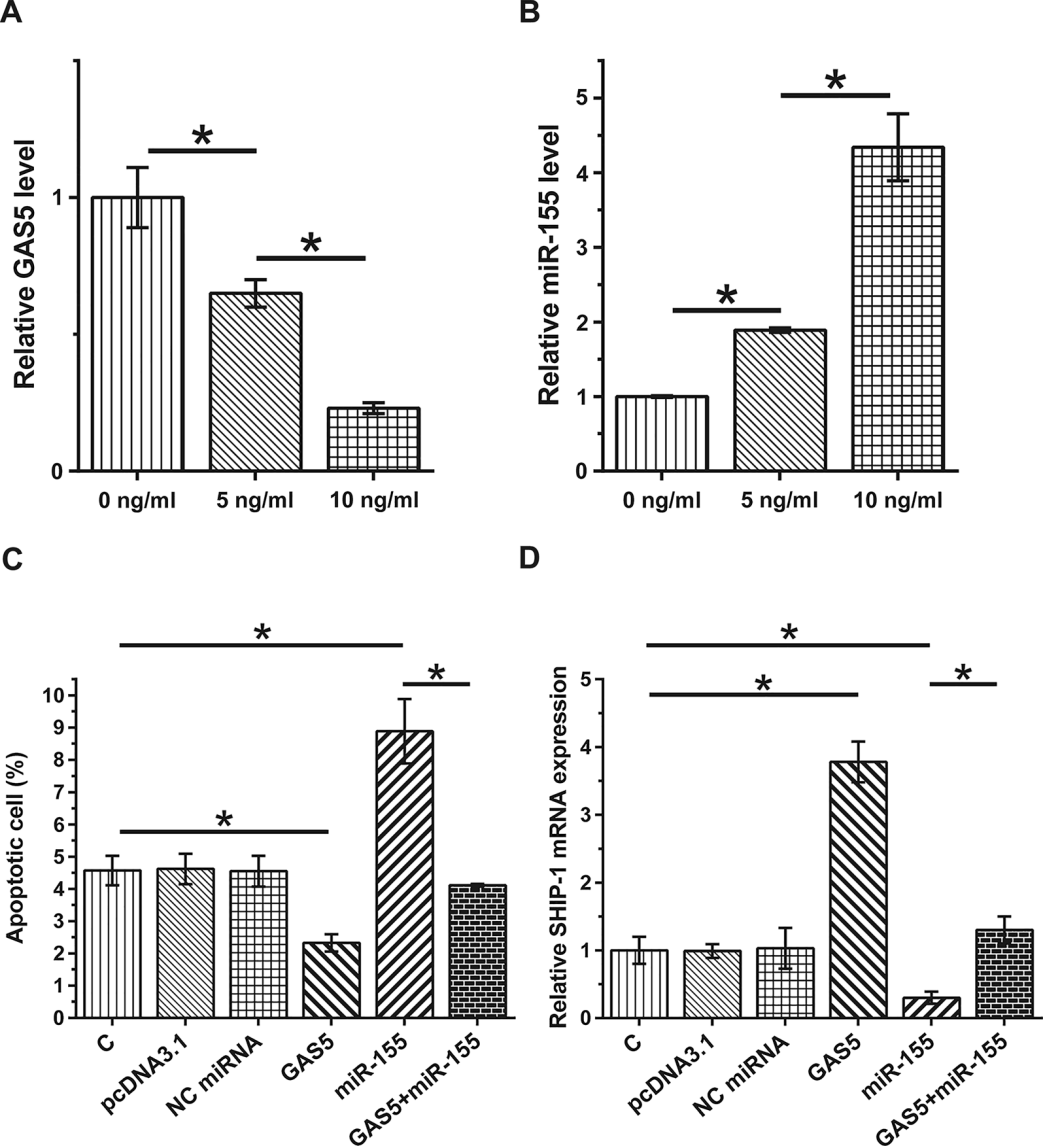
GAS5 suppressed HBEpC apoptosis through miR-155 and increased the expression of SHIP-1, a target of miR-503. HBEpCs were cultured in media containing 0, 5, and 10 ng/ml LPS for 48 h, and GAS5 (**a**) and miR-155 (**b**) expression levels were determined using RT-qPCR. Cell apoptosis assay was performed to analyze the roles of GAS5 and miR-155 in regulating HBEpC apoptosis (**c**). RT-qPCR was performed to analyze the roles of GAS5 and miR-155 in regulating SHIP-1 expression in HBEpCs (**d**). Data were derived from three independent experiments, expressed as mean ± SD, and compared by ANOVA Tukey’s test. *P < 0.05

HBEpCs were treated with 0, 5, and 10 ng/ml LPS for 48 h, and GAS5 and miR-155 expression levels were determined. LPS treatment downregulated GAS5 (Fig. 4a, *P* < 0.05) and upregulated miR-155 (Fig. 4b, *P* < 0.05). Moreover, GAS5 overexpression inhibited LPS-induced apoptosis, while miR-155 overexpression promoted cell apoptosis. In addition, GAS5 reduced the enhancing effect of miR-155 on cell apoptosis (Fig. 4c *P* < 0.05). Furthermore, TUNEL staining (Additional file 1: Fig. S1) was applied to evaluate the apoptosis level of HBEpCs transfected with indicated vectors. Similarly, the results showed that compared with HBEpCs transfected with pcDNA3.1 vector, the percentage of TUNEL-positive cells was significantly lower in HBEpCs transfected with GAS5 overexpression vector. Compared with HBEpCs transfected with NC miRNA, the percentage of TUNEL-positive cells was significantly higher in HBEpCs transfected with miR-155 mimics, and this increase was reversed in HBEpCs transfected with both miR-155 mimics and GAS5 overexpression vector. SHIP-1, a pro-inflammatory factor, has been reported to be a target of miR-155 [1, 19]. To further explore the role of GAS5 and miR-155 in inflammation, SHIP-1 expression was explored by RT-qPCR in HBEpCs pre-treated with LPS and transfected with GAS5 or miR-155 overexpression vector. As shown in Fig. 4d, GAS5 overexpression promoted LPS-induced SHIP-1 expression, while miR-155 overexpression inhibited SHIP-1 expression. In addition, GAS5 reduced the enhancing effect of miR-155 on SHIP-1 expression. These results suggested that GAS5 may downregulate miR-155 by increasing its methylation, thereby suppressing cell apoptosis and promoting SHIP-1 expression to decrease inflammation.

## Discussion

This study analyzed the interactions between GAS5 and miR-155 in pneumonia. We found that GAS5 and miR-155 expression was altered in pneumonia, and GAS5 might increase miR-155 RNA gene methylation to downregulate its expression, thereby inhibiting LPS-induced HBEpC apoptosis.

Li et al. reported that GAS5 could target miR-429/DUSP1 to suppress inflammation in alveolar epithelial cells, thereby inhibiting cell apoptosis [12]. It has been well established that lung inflammation promotes the aggregation of pneumonia [14], suggesting the potential involvement of GAS5 in pneumonia. This study is the first to report to show GAS5 downregulation in pneumonia. LPS-induced inflammatory responses and cell apoptosis promote the development of pneumonia. This study showed that LPS treatment downregulated GAS5 in HBEpCs, and GAS5 overexpression decreased the apoptotic rate of HBEpCs induced by LPS. Therefore, GAS5 may play a protective role in pneumonia, possibly by suppressing LPS-mediated cell apoptosis.

Tiwari et al. reported that increased miR-155 expression in alveolar macrophages could serve as an inflammatory marker in obese patients [13]. Many studies have investigated miR-155 changes in clinical pneumonia patients. For example, Abd-El-Fattah AA et al. found that serum miR-155 level was significantly elevated in pneumonia patients [20]. Chen CG et al. found that serum miR-155 was upregulated CAP patients than in healthy controls by using bioinformatics analysis and experimental validation. Furthermore, miR-155 inhibition attenuated LPS-induced inflammatory responses in RAW264.7 cells [21]. Similarly, our study also showed that miR-155 was also upregulated in pneumonia patients, and LPS treatment upregulated miR-155 in a dose-dependent manner. Therefore, miR-155 might participate in inflammatory responses in a LPS-dependent manner. Our study also showed the enhancing effects of miR-155 on HBEpC apoptosis induced by LPS. To our best knowledge, we, for the first time, reported that miR-155 could promote pneumonia development by increasing LPS-induced cell apoptosis and inhibiting LPS-induced SHIP-1 expression.

Interestingly, GAS5 and miR-155 play opposite roles in LPS-induced cell apoptosis and SHIP-1 expression. We also observed an inverse correlation between GAS5 and miR-155 across plasma and BALF samples from both pneumonia patients and healthy controls. In addition, GAS5 overexpression mediated the downregulation of miR-155. It is known that lncRNAs may regulate the expression of miRNAs through methylation [22]. Our study showed that GAS5 could downregulate miR-155 through methylation. However, other mechanisms may exist and need to be further explored.

Our results suggested that GAS5 expression was downregulated, and miR-155 expression was upregulated in both BALF and plasma of pneumonia patients compared with the healthy controls. These results suggested that miR-155 might be produced in the lung and subsequently transferred to plasma. Our studies on the role of GAS5 and miR-155 in pneumonia using the HBEpC model showed that GAS5 might downregulate miR-155 by increasing its methylation, thereby suppressing cell apoptosis, promoting SHIP-1 expression, and possibly decreasing inflammation. However, the specific mechanism of upregulated miR-155 expression in both BALF and plasma of pneumonia patients needed to be further studied. Macrophages, which participate in inflammatory responses, are the main cells in BALF. Wang C et al. have predicted that mir-155 could target 427 genes from mice and 674 genes from humans using the TargetScan (v.7.1) [23]. They found mir-155 mainly affects cancer pathways, B and T cell receptor signalings, neurotrophin signaling, MAPK signaling, and cell cycle signaling (PMID:28129114). They focused on the cell cycle and found that mir-155-containing macrophage exosomes suppress cardiac fibroblast proliferation, thereby promoting inflammation in cardiac fibroblasts. Similarly, we found that miR-155 promotes HBEpC apoptosis and inflammatory response, thereby promoting the development of pneumonia. Therefore, we speculated that GAS5 and miR-155 might interact in macrophages, which needs to be further studies. In addition, this study examined pediatric patients aged 1–3, an age when the adaptive immune response is developing (particularly T cell-mediated responses). Studies have shown that miR-155 level is dynamically altered during the activation of various T cell subsets, including Th1 cells, Th2 cells, Th17 cells, and Tregs [24–27]. Zhu F et al. found that miR-155 silencing attenuated DSS-induced colitis by regulating Th17/Treg cell balance and Jarid2/Wnt/β-catenin in the process [28]. Therefore, we speculated that GAS5 and miR-155 might interact in T cells subset and participate in inflammatory response through regulating Th17/Treg cell balance, which also needs to be further studied. In addition, we did not recapitulate this in an animal model of pneumonia (or LPS injury). In the future, we will detect altered miR-155 levels in lungs or plasma in mice by administration or blocking GAS5.

In conclusion, GAS5 was downregulated in pneumonia, and miR-155 was upregulated in pneumonia. GAS5 might downregulate miR-155 through methylation to suppress LPS-induced HBEpC apoptosis and promote LPS-induced SHIP-1 expression in HBEpCs.

## Supplementary Information

**Additional file 1.** Figure S1. TUNEL staining of apoptotic HBEpCs transfected with indicated vectors (40 μm). Percentage of TUNEL-positive cells = Number of positive cells/Total number counted ×100%. *P < 0.05.

## Data Availability

The data are not publicly available due to their containing information that could compromise the privacy of research participants, but are available on request from the corresponding author Yue Ma, Department of Respiratory and Critical Medicine, Clinical Medical College of Hulunbeier, Inner Mongolia University for Nationalities Hulunbuir People's Hospital, No.20 Shengli Avenue, Hulunbuir City. Email: YueMa678@163.com.
